# Comparative analysis of strand-specific RNA sequencing approaches

**DOI:** 10.1186/1753-6561-6-S6-P30

**Published:** 2012-10-01

**Authors:** Daniela Munafo, Ping Liu, Christine Sumner, Erbay Yigit, Landon Merrill, Lynne Apone, Brad Langhorst, Fiona Stewart, Eileen T Dimalanta, Theodore Davis

**Affiliations:** 1 New England Biolabs, Inc., 240 County Road, Ipswich, MA 01938, USA

## Background

Standard RNA sequencing approaches generally require double-stranded cDNA synthesis, which erases RNA strand information.

Synthesis of a randomly primed double-stranded cDNA followed by addition of adaptors for next-generation sequencing leads to the loss of information about which strand was present in the original mRNA template. The polarity of the transcript is important for correct annotation of novel genes, identification of antisense transcripts with potential regulatory roles, and for correct determination of gene expression levels in the presence of antisense transcripts. Different strand-specific RNA-seq approaches have been developed to preserve information about strand polarity with different level of performances.

## Material and methods

Using Illumina Deep Sequencing Technology, this work investigates the performance of two different directional RNA-Seq (strand-specific RNA-seq) strategies. One is based on direct ligation of adaptors to the RNA ends and the other is based on the labeling and excision of the second strand cDNA. The RNA-seq workflows present in this work have been improved over current more laborious RNA-seq methods. Their low RNA input and streamlined workflows make them compatible with high throughput and automation. We also analyze the effect of different RNA fragmentation methods (divalent cations plus heat versus enzymatic fragmentation).

## Results

We will provide a comparative full data analysis of different strand-specific RNA methods (library performance, complexity, continuity of gene coverage, strand specificity, rRNA background).

## Conclusions

Our results show improved methods for high-quality strand-specific RNA-seq library construction amenable to large-scale library construction and automation.

